# Increase of glutamate in satellite glial cells of the trigeminal ganglion in a rat model of craniofacial neuropathic pain

**DOI:** 10.3389/fnana.2023.1302373

**Published:** 2023-12-14

**Authors:** Yi Sul Cho, Won Mah, Dong Ho Youn, Yu Shin Kim, Hyoung-Gon Ko, Jin Young Bae, Yun Sook Kim, Yong Chul Bae

**Affiliations:** ^1^Department of Anatomy, Physiology and Neurobiology, School of Dentistry, Kyungpook National University, Daegu, Republic of Korea; ^2^Department of Oral & Maxillofacial Surgery, School of Dentistry, Programs in Integrated Biomedical Sciences, Translational Sciences, Biomedical Engineering, Radiological Sciences, University of Texas Health Science Center at San Antonio, San Antonio, TX, United States

**Keywords:** satellite glial cell, trigeminal, orofacial, glutamate, neuropathic pain

## Abstract

**Introduction:**

Satellite glial cells (SGCs) that envelop the cell bodies of neurons in sensory ganglia have been shown to both release glutamate, and be activated by glutamate in the context of nociceptive signaling. However, little is known about the subpopulations of SGCs that are activated following nerve injury and whether glutamate mechanisms in the SGCs are involved in the pathologic pain.

**Methods:**

To address this issue, we used light and electron microscopic immunohistochemistry to examine the change in the glutamate levels in the SGCs and the structural relationship between neighboring neurons in the trigeminal ganglion (TG) in a rat model of craniofacial neuropathic pain, CCI-ION.

**Results:**

Administration of ionomycin, ATP and Bz-ATP induced an increase of extracellular glutamate concentration in cultured trigeminal SGCs, indicating a release of glutamate from SGCs. The level of glutamate immunostaining in the SGCs that envelop neurons of all sizes in the TG was significantly higher in rats with CCI-ION than in control rats, suggesting that SGCs enveloping nociceptive as well as non-nociceptive mechanosensitive neurons are activated following nerve injury, and that the glutamate release from SGCs increases in pathologic pain state. Close appositions between substance-P (SP)-immunopositive (+) or calcitonin gene-related peptide (CGRP)+, likely nociceptive neurons, between Piezo1+, likely non-nociceptive, mechanosensitive neurons and SP+ or CGRP+ neurons, and between SGCs of neighboring neurons were frequently observed.

**Discussion:**

These findings suggest that glutamate in the trigeminal SGCs that envelop all types of neurons may play a role in the mechanisms of neuropathic pain, possibly via paracrine signaling.

## Introduction

Satellite glial cells (SGCs) that envelop cell bodies (somata) of neurons in the sensory ganglia have been shown to contribute to neuronal sensitization and pathologic pain through SGC-SGC and SGC-neuron communication via gap junctions and ATP signaling. Thus, the expression of the gap junction protein Cx34, the coupling of SGCs or neurons by gap junctions, the release of ATP from SGCs, and the sensitivity of SGCs to ATP are all significantly increased following nerve injury and inflammation, leading to neuronal hyperexcitability (Blum et al., 2014; Kim et al., 2016; Shinoda et al., 2019; Hanani and Spray, 2020).

The existence of SGC-mediated glutamate signaling in dorsal root ganglia (DRG) and trigeminal ganglia (TG) is supported by studies that show that SGCs contain and release glutamate when stimulated by the calcium ionophore ionomycin and by potassium chloride (Laursen et al., 2014; da Silva et al., 2015), and that SGCs are activated by glutamate or glutamate receptor agonists (Castillo et al., 2013; Kung et al., 2013; Ferrari et al., 2014).

The role of glutamate in pain transduction is apparently multifaceted and, after more than 30 years of research, still insufficiently well understood. Aside from its role as a neurotransmitter in the synapses of spinal and trigeminal primary afferents, glutamate is thought to participate in the paracrine signaling between the cell bodies of neurons and glia, and injecting glutamate into the TG or DRG induces muscle pain and mechanical hyperalgesia (Ferrari et al., 2014; Laursen et al., 2014). It was also shown that nerve injury induces glutamate release from astrocytes in the spinal cord, contributing to neuropathic pain (Ji et al., 2016). However, so far, there is little evidence implicating glutamate in the SGCs in the neuropathic pain following peripheral nerve injury.

To provide additional information on this issue, we examined the change of glutamate levels in the trigeminal SGCs in a rat model of craniofacial neuropathic pain by electron microscopic postembedding immunogold labeling and quantitative analysis. We also studied the spatial relationship between trigeminal SGCs of neighboring neurons and between nociceptive neurons and nociceptive or mechanosensitive neurons, which may facilitate paracrine communication that can contribute to hyperalgesia and/or allodynia.

## Materials and methods

### Animals and tissue preparation

All experimental procedures were approved by the Intramural Animal Care and Use Committee at the Kyungpook National University, and followed the guidelines of the National Institute of Health. Twenty male Sprague–Dawley rats (170–190 g) were used for this study: Three rats for light microscopic (LM) immunohistochemistry, three rats for routine electron microscopy, eight rats for a behavioral assay for mechanical allodynia after chronic constriction injury of infraorbital nerve (CCI-ION), followed by electron microscopic (EM) postembedding immunohistochemistry, and six rats for a glutamate release assay.

### Surgery for CCI-ION

Rats were anesthetized with intraperitoneal injection of a mixture of ketamine (40 mg/kg) and xylazine (4 mg/kg), and surgery to induce CCI-ION was performed according to the original protocol (Imamura et al., 1997). Briefly, a one-centimeter incision was made along the gingivo-buccal margin, next to the first upper molar. Approximately 5 mm segment of the infraorbital nerve was isolated from the surrounding tissue, and two 5–0 chromic gut (C540, AILEE Co., LTD, Busan, Korea) ligatures were tied loosely around it. The incision was closed with two 4–0 silk sutures. In sham-operated animals, the surgery was identical, except that no ligatures were placed around the infraorbital nerve.

### Behavioral assay for mechanical allodynia

Rats were habituated for 30 min to a cage in a darkened and noise-free room. Withdrawal responses were measured after 10 trials of 4-s duration in 10-s intervals of constant air-puff pressure, as described previously (Ahn et al., 2009). The response threshold was determined as the air-puff pressure at which the rat responded in 5 of the 10 trials. The cut-off pressure was 40 psi.

### Light microscopic immunohistochemistry

For immunofluorescence, naïve rats were deeply anesthetized with sodium pentobarbital (80 mg/kg, i.p.) and perfused intracardially with 100 mL heparinized 0.9% saline, followed by 500 mL freshly-prepared fixative, containing 4% paraformaldehyde (PFA) in 0.1 M phosphate buffer at pH 7.4 (PB). Trigeminal ganglions (TGs) were dissected out and postfixed in the same fixative for 2 h at 4°C, then cryoprotected in 30% sucrose in PB overnight at 4°C. The next day, 30-μm-thick sections were cut on a freezing microtome. Sections were treated with 50% ethanol in 10% normal donkey serum (NDS, Jackson ImmunoResearch, West Groove, PA) for 30 min, and incubated overnight in a rabbit anti-substance P (SP, 1:2,000; 20064, Immunostar, WI) or a rabbit anti-calcitonin gene-related peptide (CGRP, 1:5000; 24112, Immunostar) antibody alone or in combination of guinea pig anti-SP (1:1,000; AB5892, Chemicon, CA) or goat anti-CGRP antibody (1,500; AB36001, Chemicon) with rabbit anti-Piezo1 antibody (1,300; APC087, Alomone Labs, Jerusalem). On the next day, the sections were incubated with a Cy3-conjugated donkey anti-rabbit antibody alone or a combination of a Cy3-conjugated donkey anti-guinea pig or donkey anti-goat antibody and a FITC-conjugated donkey anti-rabbit antibodies (1,200, in PB, Jackson ImmunoResearch) for 3 h. Sections were mounted on slides, coverslipped with Vectashield (Vector Laboratories, Burlingame, CA), and examined on a Zeiss Axioplan 2 fluorescence microscope. Micrographs were obtained with an Exi digital camera (Q-imaging Inc., Surrey, CA), attached to the microscope. Images were converted to grayscale and brightness and contrast were enhanced using identical adjustment steps for all images. The threshold level for defining neurons as immunopositive was determined at 100–120 gray level in images with 256 gray levels using Image J software (NIH, Bethesda, MD).

The specificity of the immunostaining was evaluated by omission of the primary or secondary antibodies, which completely eliminated the specific staining. Specific immunostaining with SP and Piezo1 was also completely abolished by preadsorption with the respective peptides (SP: S6883, Sigma-Aldrich, 10 μg/mL; Piezo1: BLP-PC087, Alomone Labs, 8 μg/mL).

### Electron microscopic immunohistochemistry

On day 22 after surgery, when mechanical allodynia is most prominent, rats of the sham-operated and CCI-ION groups, as well as naïve rats, were deeply anesthetized with sodium pentobarbital (80 mg/kg, i.p.) and perfused intracardially with 100 mL heparinized 0.9% saline, followed by 500 mL freshly-prepared fixative, containing 1% PFA and 2.5% glutaraldehyde in 0.1 M PB. TGs were dissected out and fixed in the fixative used for perfusion for an additional 2 h at 4°C. Sections were cut at 50 μm on a Vibratome and osmicated in 1% osmium tetroxide in PB for 30 min. After that, sections were dehydrated in graded ethanol, flat-embedded in Durcupan ACM (Fluka, Buchs, Switzerland) between Aclar plastic films (EMS, Hatfield, PA), and cured for 48 h at 60°C. Small chips containing the ophthalmo-maxillary region of the TG were cut out of the wafers and glued onto blank resin blocks with cyanoacrylate. Thin sections were cut with a diamond knife, mounted on formvar-coated single slot nickel grids, and stained with uranyl acetate and lead citrate. Grids were examined on a Hitachi H7500 electron microscope (Hitachi, Tokyo, Japan) at 80 kV accelerating voltage. Images were captured with Digital Micrograph software driving a cooled CCD camera (SC1000; Gatan, Pleasanton, CA) attached to the microscope, and saved as TIFF files.

### Postembedding immunogold staining

Postembedding immunogold labeling for glutamate was performed according to the method described previously by our laboratory (Paik et al., 2012, 2021). Briefly, grids were treated for 6 min in 1% periodic acid, to etch the resin, and for 10 min in 9% sodium periodate, to remove the osmium tetroxide. Grids were then transferred to tris-buffered saline containing 0.1% triton X-100 (TBST; pH 7.4) for 10 min. Grids were further incubated with rabbit polyclonal antiserum against glutamate (1:80,000; G6642, Sigma-Aldrich; RRID: AB_259946) in TBST containing 2% human serum albumin for 4 h at room temperature. To eliminate cross-reactivity, the diluted antiserum was preadsorbed overnight with glutaraldehyde (G)-conjugated amino acids (600 μM glutamine-G, 200 μM β-alanine-G, and 100 μM aspartate-G; Ottersen et al., 1986). After rinsing in TBST, grids were incubated in goat anti-rabbit IgG antibody coupled to 15 nm gold particles (1:25 in TBST containing 0.05% polyethylene glycol; BioCell, Cardiff, United Kingdom; RRID: AB_1769134) for 2 h at room temperature. Grids were counterstained with uranyl acetate and lead citrate, and examined with the electron microscope, images were captured and saved as above.

The specificity of the immunolabeling was evaluated by omission or replacement of the primary antiserum with normal rabbit serum or by preadsorption of the diluted anti-glutamate serum with 300 μM glutamate-G, which abolished the specific immunostaining. It was also confirmed on “sandwiches” of rat brain macromolecule-glutaraldehyde fixation complexes of GABA, glutamate, taurine, glycine, aspartate and glutamine (Ottersen, 1987; Paik et al., 2007), as routinely practiced in our laboratory.

### Quantitative analysis

We divided the TG neurons into three groups, based on the size of their somata: small (<400 μm^2^ in cross-sectional area), medium (400–800 μm^2^), and large (>800 μm^2^; Bae et al., 2018). Twelve TG neurons of each group, together with their enveloping SGCs, in each of 4 rats with CCI-ION, and 4 sham-operated rats, were used for quantitative analysis. The density of gold particles coding for glutamate (number of gold particles/μm^2^) was measured in 80–110 electron micrographs from each of 3 small, 3 medium-sized, and 3 large neuronal somata in each TG using a digitizing tablet and Image J software (NIH, Bethesda, MD). The density of gold particles was determined as the number of gold particles in randomly-selected rectangular areas of 4 μm^2^ of the cytoplasm of each neuron (6, 9 and 12 rectangular areas per small, medium-sized, and large neurons, respectively, or a total area of 24–48 μm^2^ per neuron) and in their enveloping SGCs (3–12 randomly-selected areas of the cytoplasm, or a total area of 5–50 μm^2^ per SGC). The gold particles over the nucleus and the mitochondria were excluded from analysis. The density of gold particles over extracellular areas was used to normalize for different background density in different sections and different immunohistochemical runs.

### SGC culture and glutamate release assay

Rats, anesthetized with intraperitoneal injection of ketamine (40 mg/kg) and xylazine (4 mg/kg), were decapitated and the heads were transferred to ice-cold DPBS. The TGs were dissected out, separated from the surrounding dura, and transferred to ice-cold HBSS. TGs were then minced with a sterile razor blade, placed in a collagenase solution (5 mg/mL in HBSS; C9891, Sigma-Aldrich), and incubated for 20 min at 37°C, shaking every 5 min. After centrifugation at 1,300 rpm for 5 min, the pellet was dissociated with 1 mL of 0.125% trypsin (15090–046, Gibco, MA), and incubated for 10 min at 37°C. After another centrifugation at 1,300 rpm for 5 min, the pellet was mechanically dissociated with a Pasteur pipette, placed in DMEM containing 10% FBS, and incubated in 5% CO2 incubator at 37°C. The DMEM medium was changed every 3 days. After 3 weeks, cells were plated into a 12-well culture plate at 5×10^5^ cells per well.

Cultured SGCs were tested using a glutamate assay kit (ab83389, Abcam, Cambridge, United Kingdom) according to the manufacturer’s protocol. Briefly, the cells were treated with an extracellular solution (in mM, 140 NaCl, 5 KCl, 2 CaCl_2_, 1 MgCl_2_, 10 HEPES, 10 glucose) containing either 0.125% DMSO as a vehicle or 5 μM Ionomycin, 200 μM ATP, and 100 μM Bz-ATP, and 50 μL of the solution was harvested into a 96-well plate at 5 min, 15 min, and 30 min post treatment. The absorbance at 450 nm wavelength was measured with a plate reader (iMark, Biorad, CA).

### Statistical analysis

**Figure 1:**
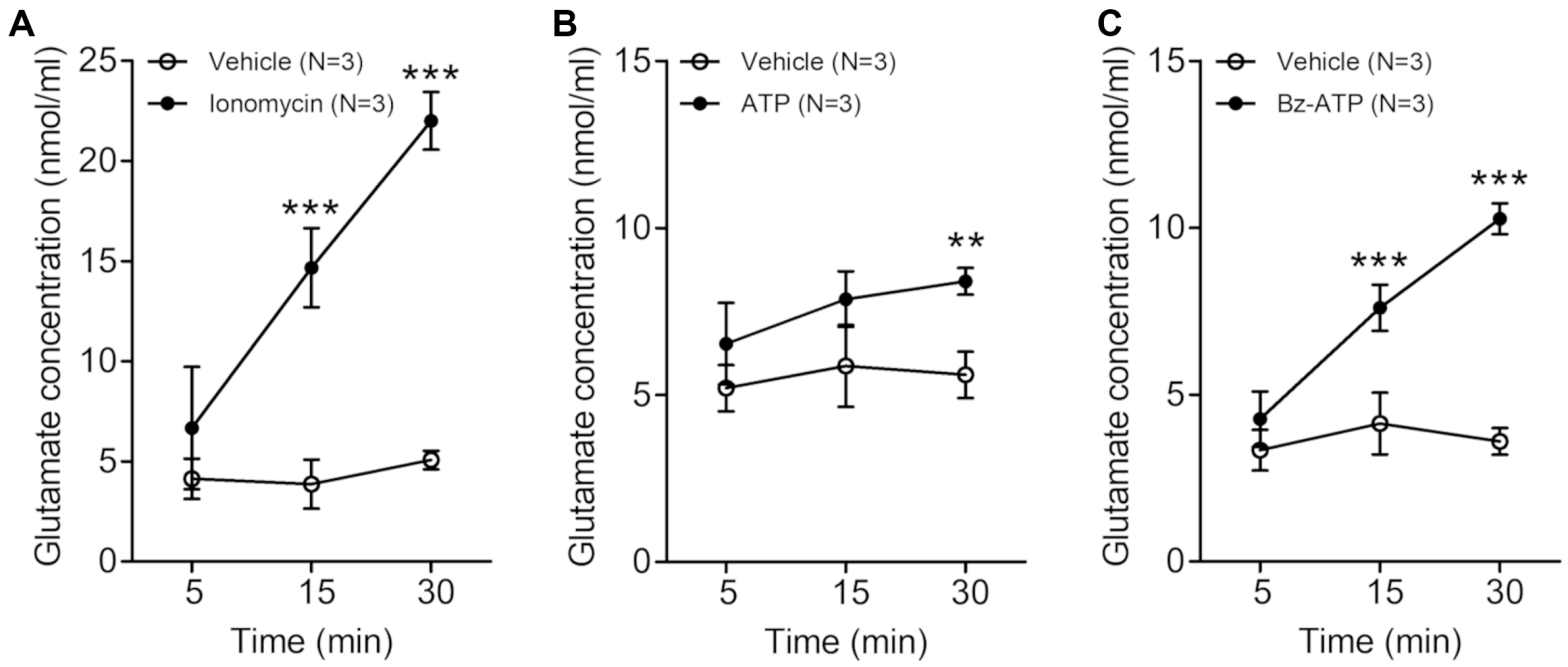
Line graphs **(A–C)** showing extracellular glutamate concentration (nmol/ml, mean ± SD) in trigeminal satellite glial cells culture at various time points after treatment with 5 μM ionomycin **(A)**, 200 μM ATP **(B)** or 100 μM Bz-ATP **(C)**. The extracellular glutamate concentration is significantly higher in the cultures treated with ionomycin, ATP and Bz-ATP than in the cultures treated with vehicle. *N* = 3 rats in each group. **p < 0.01, ***p < 0.001, two-way ANOVA, Bonferroni *post-hoc* test.

**Figure 2:**
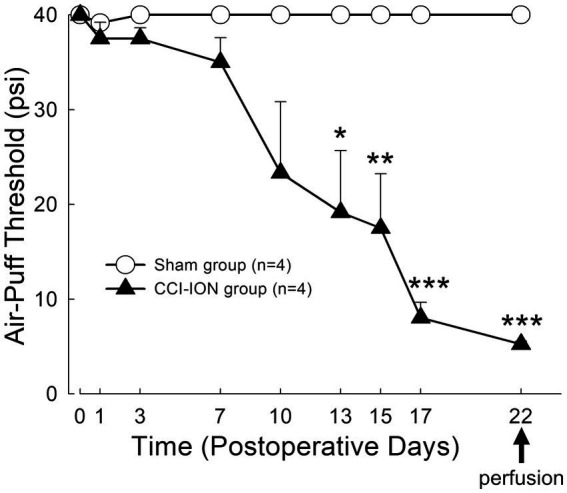
Time course of changes in air-puff threshold following chronic constriction injury of the infraorbital nerve (CCI-ION) in rats: Air-puff threshold was rarely altered in sham-operated rats. However, it was significantly decreased from day 13 following CCI-ION until day 22 (sham vs. CCI-ION: 40.00 ± 0.00 vs. 19.17 ± 6.51 psi, *p* = 0.015 in day 13 after surgery, 40.0 ± 0.0 vs. 5.25 ± 0.30, *p* < 0.001 in day 22 after surgery. Values are mean ± SEM) when mechanical allodynia was most prominent, and when rats were sacrificed to evaluate glutamate level in the satellite glial cells and neurons. *N* = 4 rats in each group. *p < 0.05, **p < 0.01, ***p < 0.001, sham vs. CCI-ION group, two-way ANOVA, Bonferroni *post-hoc* analysis.

**Figure 3:**
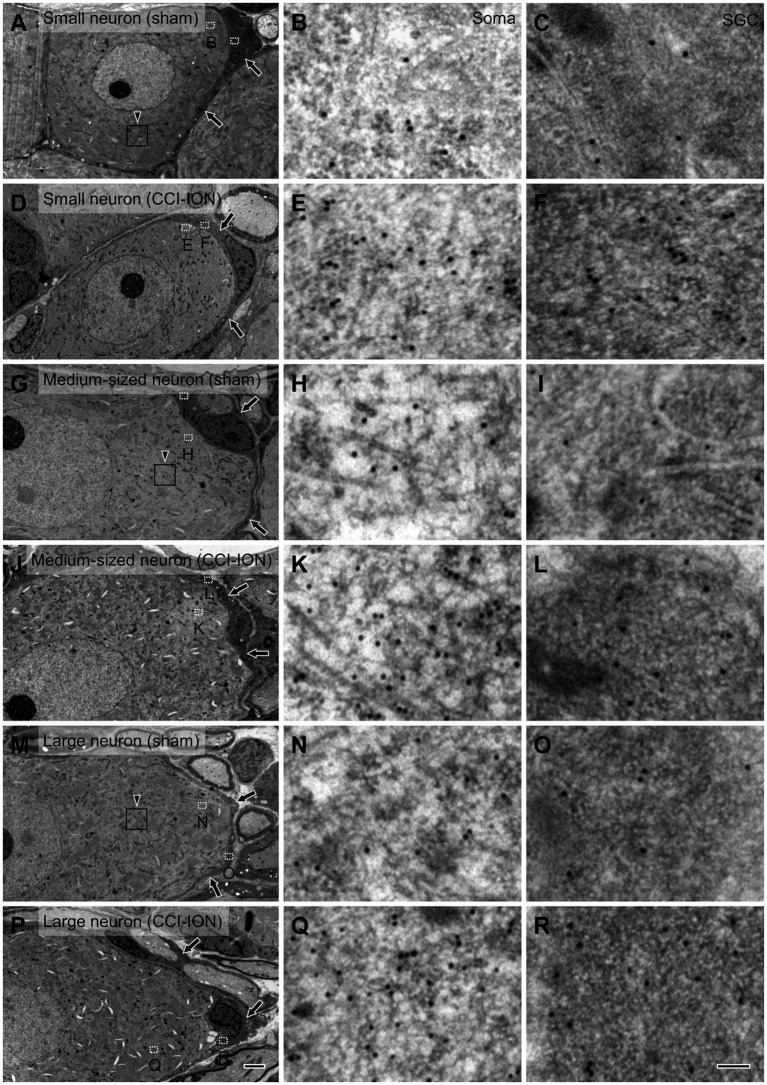
Electron micrographs of the trigeminal ganglion showing immunogold labeling for glutamate in small **(A–F)**, medium-sized **(G–L)** and large **(M–R)** neurons and their satellite glial cells (SGCs, arrows) in sham **(A–C,G-I,M–O)** and CCI-ION **(D–F,J–L,P–R)** rats; gold particles coding for glutamate are denser in the CCI-ION than in the sham-operated rats. **(B,C,E,F,H,I,K,L,N,O,Q,R)** are enlargements of the boxed areas (outlined with dotted line) in **A,D,G,J,M,P** in the neuron and the SGC, respectively. Arrowheads in **A,G,M** indicate rectangular area of 4 μm^2^ for analyzing density of gold particles: We measured gold particle density in 6, 9, 12 rectangular areas per a small, medium-sized and large neurons, respectively. Scale bars = 2 μm in **P** **(**also applies to **A,D,G,J,M)**; 100 nm in **R (** also applies to **B,C,E,F,H,I,K,L,N,O,Q)**.

Data were presented as mean ± SD (extracellular glutamate concentration in Figure 1) or mean ± SEM (air-puff threshold in Figure 2 and normalized value of gold particle density in Figure 3). Statistical analysis was performed using SPSS v.21.0 software (SPSS Inc., Chicago, IL). Difference between groups in extracellular glutamate concentration and in air-puff threshold was examined using two-way ANOVA, followed by Bonferroni post-hoc test. Even though sample size for analysis of extracellular glutamate concentration (in Figure 1) is small (*n* = 3 per group) power analysis showed 90% power at *p* < 0.05. Difference between groups in normalized value of gold particle density was examined using unpaired student *t*-test: Interanimal variability in normalized value of gold particle density within the same group was insignificant (one-way ANOVA), and the data could be pooled per group for analysis. Values of *p* < 0.05 were considered statistically significant. The correlation of glutamate density in each neuron to that in its enveloping SGC was studied using Pearson correlation analysis.

## Results

### Stimulation of SGCs induced glutamate release *in vitro*

In the trigeminal SGCs culture, the level of extracellular glutamate increased significantly 15–30 min after treatment with the calcium-ionophore ionomycin (vehicle vs. ionomycin: 3.87 ± 1.22 vs. 14.67 ± 1.97 nmoL/mL, *p* = 0.001 at 15 min, 5.07 ± 0.46 vs. 22.00 ± 1.44 nmoL/mL, *p* < 0.001 at 30 min after treatment with ionomycin. Values are mean ± SD, two-way ANOVA), ATP (vehicle vs. ATP: 5.60 ± 0.69 vs. 8.40 ± 0.40 nmoL/mL, *p* = 0.004 at 30 min after ATP treatment), and Bz-ATP (vehicle vs. Bz-ATP: 4.13 ± 0.92 vs. 7.60 ± 0.69 nmoL/mL, *p* = 0.007 at 15 min, 3.60 ± 0.40 vs. 10.27 ± 0.46 nmoL/mL, *p* < 0.001 at 30 min after Bz-ATP treatment), suggesting that SGCs, when stimulated, release glutamate (Figure 1).

### Rats with chronic constriction injury of the infraorbital nerve manifested mechanical allodynia

Behavioral analysis demonstrated that chronic constriction injury of the infraorbital nerve (CCI-ION) produces prolonged mechanical allodynia in the facial area innervated by the infraorbital nerve (whisker pad). Sham-operated rats rarely showed alteration in air-puff thresholds. However, CCI-ION rats showed significant decrease in air-puff threshold on day 13 (sham vs. CCI-ION: 40.00 ± 0.00 vs. 19.17 ± 6.51 psi, *p* = 0.015, Values are mean ± SEM, two-way ANOVA), which persisted until day 22 (sham vs. CCI-ION: 40.00 ± 0.00 vs. 5.25 ± 0.31 psi, *p* < 0.001), when mechanical allodynia was most prominent, and when we sacrificed the animals for analysis of glutamate level in trigeminal neurons and their enveloping SGCs (Figure 2).

### SGCs exhibited elevated levels of glutamate following CCI-ION

Since its introduction more than 30 years ago, the method of assessing the concentration of glutamate in the CNS by determining the density of immunogold particles in cellular and subcellular structures, such as cell bodies and axon terminals, has been applied successfully by many, including our laboratory (Ottersen, 1989a, b; Shupliakov et al., 1992; Paik et al., 2021). Moreover, since the cytoplasm of SGCs is more electron-dense than that of neurons, it is also possible to distinguish the two cell types and compare the concentration of glutamate in them to that in the extracellular matrix.

**Figure 4:**
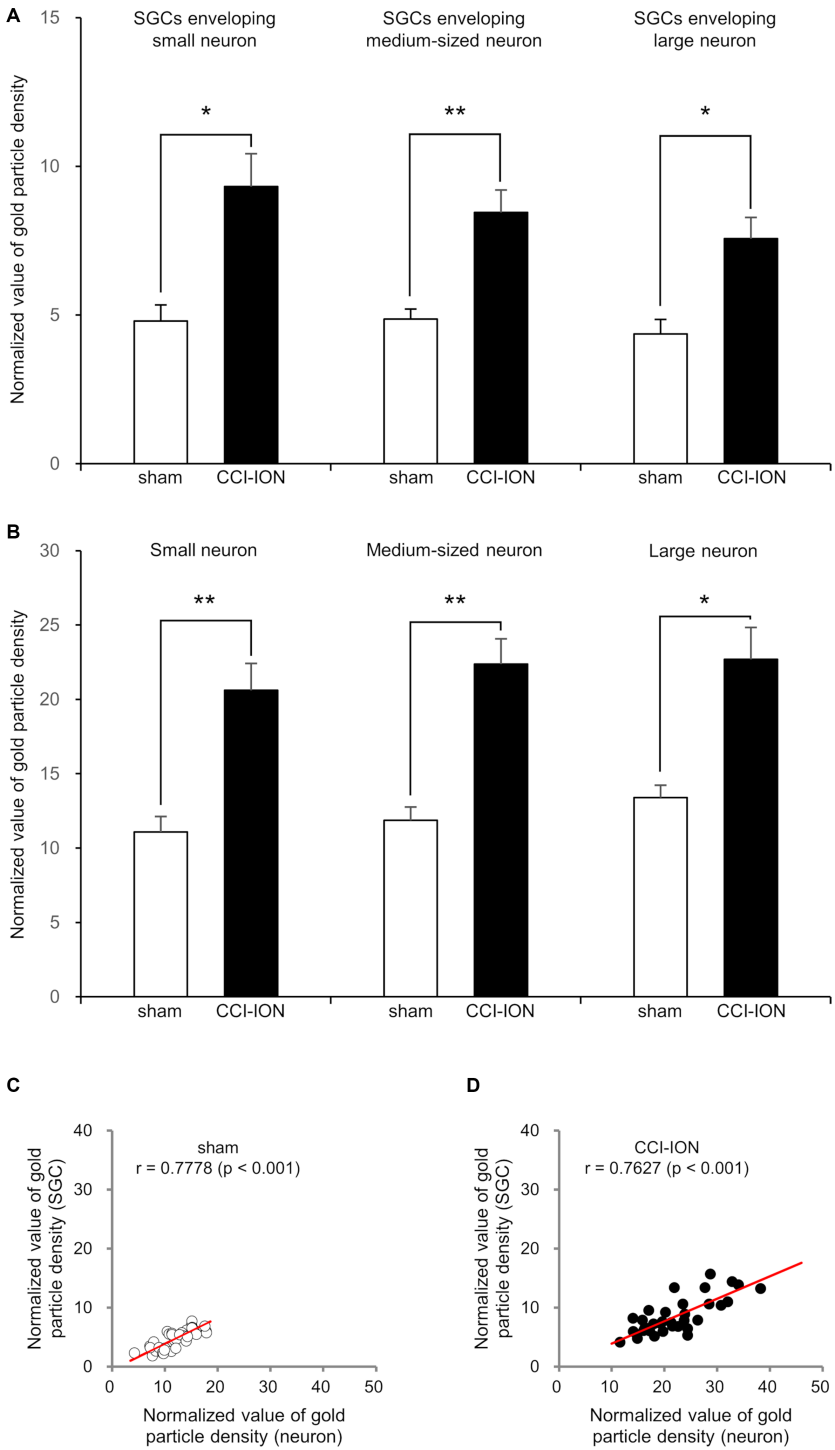
Normalized value (Mean ± SEM) of the density of gold particles coding for glutamate in satellite glial cells (SGCs, **A**) and in the trigeminal neurons they envelop **(B)**, and scatterplots of the normalized values of the density of gold particles coding for glutamate in SGCs and in the trigeminal neurons they envelop **(C,D)** in rats with CCI-ION vs. sham-operated rats. **(A,B)** Gold particle density for glutamate in SGCs and the neurons they envelop is significantly higher in the CCI-ION rats than in sham-operated rats. **(C,D)** Gold particle density for glutamate in SGCs is positively correlated to that in the neurons they envelop. *N* = 12 in each small, medium-sized, and large neurons from 4 rats in each group. *p < 0.01, **p < 0.001, unpaired student t-test for comparison of normalized values of gold particle density. Pearson correlation analysis for correlation of the normalized values of gold particle density.

In SGCs that envelop neurons of all sizes (small, medium-sized, and large), the normalized values of the density of gold particles coding for glutamate was significantly higher in the CCI-ION group than in the control group (sham vs. CCI: 4.80 ± 0.55 vs. 9.32 ± 1.11, *p* = 0.002 in SGCs enveloping small soma, 4.86 ± 0.34 vs. 8.45 ± 0.75, *p* < 0.001 in SGCs enveloping medium-sized soma, 4.36 ± 0.49 vs. 7.57 ± 0.70, *p* = 0.001 in SGCs enveloping large soma, unpaired student *t*-test. Values are mean ± SEM, Figures 3,4). Also in neurons of all sizes, the normalized values of the gold particle density was significantly higher in the CCI-ION group than in controls (sham vs. CCI: 11.07 ± 1.05 vs. 20.61 ± 1.81, *p* < 0.001 in small soma, 11.88 ± 0.89 vs. 22.37 ± 1.71, *p* < 0.001 in medium-sized soma, 13.39 ± 0.85 vs. 22.69 ± 2.14, *p* = 0.001 in large soma, unpaired student *t*-test. Values are mean ± SEM, Figures 3,4). The density of gold particles in SGCs was positively correlated with that in the neurons they envelop in both CCI-ION and control groups (correlation coefficient *r* = 0.7778 in sham group, *r* = 0.7627 in CCI-ION group, Pearson correlation analysis; Figure 4).

### Close apposition between presumed nociceptive and nociceptive or non-nociceptive neurons and between two SGCs of neighboring neurons was frequently observed

**Figure 5:**
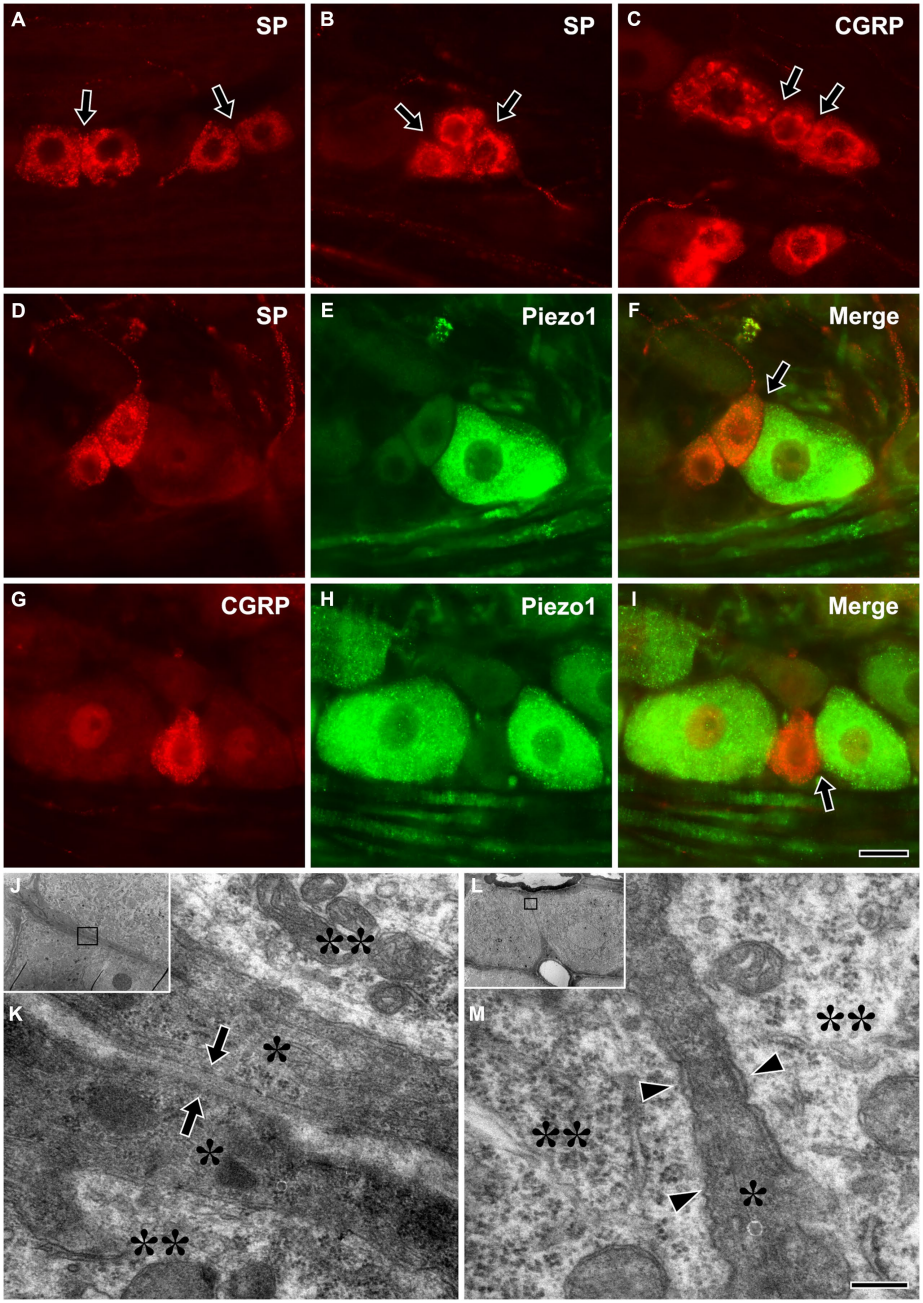
Light micrographs showing immunofluorescent staining for SP **(A,B)** or CGRP **(C)** alone, and in combination with Piezo1 **(D–I)** in the trigeminal ganglion, and electron micrographs showing a close apposition between SGCs of nearby trigeminal neurons **(J,K)**, and nearby neurons that share a single SGC **(L,M)**. **(A–I)** Close appositions (arrows) between small SP+ **(A,B)** or CGRP+ **(C)**, likely nociceptive neurons, and between large Piezo1+, likely non-nociceptive, mechanosensitive neurons and small SP+ **(D–F)** or CGRP+ neurons **(G–I)**. Although SGCs are not observed in these light micrographs, the neurons are actually closely apposed through their enveloping SGCs. **(J–M)** Close apposition with a ~ 50 nm wide gap (arrows in **K**) between SGCs of nearby neurons **(J,K)**, and nearby neurons sharing a single SGC **(L,M)** were frequently observed in the TG. **(K,M)** are enlargements of the boxed areas in **(J,L)**, respectively. Asterisks and double asterisks in **(K,M)** indicate the SGCs and the neurons they envelop, respectively. Arrowheads in **M** indicate a narrow cleft between the neurons and the SGC. Light and electron microscopic observations were performed on the sections of 3 rats, respectively. Scale bar = 50 μm in **I** (also applicable to **A–H**) and 200 nm in **M** (also applicable to **K**).

Immunofluorescent staining for SP or CGRP alone or in combination with Piezo1 revealed frequent close apposition between small SP+ or CGRP+ (likely nociceptive), and between large Piezo1+ (likely non-nociceptive, mechanosensitive) neurons and small SP+ or CGRP+ neurons (Figures 5A–I). Electron microscopic examination of serial sections showed that, in some parts of the interface between SGCs of nearby neurons, they were only separated by a ~ 50 nm wide gap, similar to the synaptic cleft of the neuromuscular junction (Gabbiani and Cox, 2010). In addition, closely apposed neurons sharing a single SGC were also frequently observed (Figures 5J–M).

## Discussion

The main findings of the present study are that glutamate in the trigeminal SGCs that envelop neurons of all sizes increased following peripheral nerve injury, and that close appositions between SGCs of nearby nociceptive and non-nociceptive or nociceptive neurons, were frequent. These findings suggest that glutamate in the SGCs that envelop neurons of all types including nociceptive and non-nociceptive mechanosensitive neurons may play a role in the mechanisms of neuropathic pain, possibly via paracrine communication between SGCs that envelop nearby nociceptive neurons, and between SGCs of non-nociceptive, mechanosensitive neurons and nearby nociceptive neurons in the TG.

That the levels of glutamate in the trigeminal neurons of all sizes increased after CCI-ION is consistent with previous reports that (1) glutamate increases in the DRG and TG neurons following peripheral nerve injury and inflammation (Kung et al., 2013; Hoffman et al., 2016), (2) the spontaneous activity and excitability of neurons of all types in the DRG is increased following nerve injury (Song et al., 2003; Sapunar et al., 2005; Djouhri et al., 2012), and (3) the release of glutamate from central terminals of sensory fibers of all types is increased following CCI-ION (deGroot et al., 2000; Dmitrieva et al., 2004; Kumar et al., 2013; Kung et al., 2013). However, our results also suggest that glutamate release from the somata of all neuronal types in the TG is increased following nerve injury, possibly leading to activation of their SGCs, corroborating previous reports that stimulation of DRG neurons evokes glutamate release from their somata (Gu et al., 2010; Kung et al., 2013), and that SGCs are activated by glutamate and by glutamate receptor agonists (Castillo et al., 2013; Kung et al., 2013; Ferrari et al., 2014).

SGCs express glutamate receptors (Ivanusic et al., 2011; Castillo et al., 2013; Kung et al., 2013; Boye Larsen et al., 2014; Ferrari et al., 2014; Wagner et al., 2014) and injecting glutamate or the glutamate receptor agonist NMDA into the TG or DRG induces muscle pain or hyperalgesia (Ferrari et al., 2014; Laursen et al., 2014), suggesting that SGCs can be activated by glutamate. Trigeminal SGCs express glutamate aspartate transporter (GLAST) and glutamate transporter 1 (GLT-1) that is involved in the clearance of extracellular glutamate (Carozzi et al., 2011). Expression of astrocytic GLAST and GLT-1 and astrocytic uptake of glutamate is reduced following nerve injury (Sung et al., 2003; Xin et al., 2009; Nie and Weng, 2010). In addition, reducing GLAST expression in the TG using GLAST-dsRNA results in facial allodynia GLAST (Ohara et al., 2009). Given these reports, it is possible to assume that increase in glutamate level in the SGCs following nerve injury can be induced by down regulation of GLAST or GLT-1 and reduction of perineuronal glutamate clearance leading to activation of glutamate receptors on SGCs. The treatment of trigeminal SGC culture with ionomycin, ATP and Bz-ATP showed significant increase of extracellular glutamate, suggesting that SGCs, when activated, release glutamate. It was shown that SGCs are activated and release cytokine and ATP following nerve injury and inflammation (Kushnir et al., 2011; Hanani and Spray, 2020). Thus, it is also expected that the extracellular glutamate concentration in SGCs culture of CCI-ION following stimulation may be higher than naïve rats. Glutamate levels in the SGCs that envelop trigeminal neurons of all sizes increased in our model of neuropathic pain. Taken together, our results suggest that glutamate release from SGCs that envelop nociceptive as well as non-nociceptive, mechanosensitive neurons may be increased following peripheral nerve injury, which can contribute to the development of pathologic pain.

Careful analysis of the ultrastructural morphology of TG in serial sections revealed frequent close appositions between SGCs of nearby neurons with a ~ 50 nm gap between the cell membranes. This structure, which appeared similar to the synaptic cleft of neuromuscular junctions (Gabbiani and Cox, 2010), may allow diffusion of glutamate from the SGC to activate glutamate receptors in the apposed cell, and seems well suited to facilitate paracrine communication between SGCs and SGCs and/or nearby neurons. It may also subserve the communication between neighboring nociceptive neurons or between neighboring nociceptive and non-nociceptive, mechanosensitive neurons in the TG, which can contribute to the mechanisms of hyperalgesia and allodynia, respectively.

In summary, our findings suggest that glutamate release from SGCs that envelop nociceptive and non-nociceptive neurons may increase following peripheral nerve injury, which can contribute to the development of pathologic pain via paracrine communication between SGCs of nearby nociceptive or non-nociceptive, mechanosensitive neurons. Further study analyzing expression of glutamate receptor in SGCs, it is modulation after CCI-ION and effect of blocking glutamate receptor in SGCs on pathologic pain is needed to clarify involvement of SGCs-associated glutamate signaling in the pathologic pain.

## Data availability statement

The original contributions presented in the study are included in the article/supplementary material, further inquiries can be directed to the corresponding author.

## Ethics statement

The animal study was reviewed and approved by the Intramural Animal Care and Use Committee at the Kyungpook National University, and followed the guidelines of the National Institute of Health. The study was conducted in accordance with the local legislation and institutional requirements.

## Author contributions

YC: Conceptualization, Writing – review & editing, Investigation. WM: Conceptualization, Writing – review & editing, Methodology. DY: Conceptualization, Writing – review & editing. YuK: Conceptualization, Formal analysis, Writing – original draft. H-GK: Data curation, Formal analysis, Writing – review & editing. JB: Formal analysis, Investigation, Methodology, Writing – review & editing. YunK: Conceptualization, Writing – review & editing, Formal analysis. YB: Conceptualization, Supervision, Writing – original draft, Writing – review & editing.
